# Assessing the bioconfinement potential of a *Nicotiana* hybrid platform for use in plant molecular farming applications

**DOI:** 10.1186/1472-6750-13-63

**Published:** 2013-08-06

**Authors:** J Hollis Rice, Richard E Mundell, Reginald J Millwood, Orlando D Chambers, C Neal Stewart, H Maelor Davies

**Affiliations:** 1 Department of Plant Sciences, University of Tennessee, Knoxville, TN 37996, USA; 2 Kentucky Tobacco Research & Development Center, University of Kentucky, Lexington, KY 40546, USA; 3 Department of Plant & Soil Sciences, University of Kentucky, Lexington, KY 40546, USA

**Keywords:** Gene flow, Male-sterility, Pharming, Bioconfinement, *Nicotiana*, Green fluorescent protein (GFP), Plant-made-pharmaceuticals (PMPs)

## Abstract

**Background:**

The introduction of pharmaceutical traits in tobacco for commercial production could benefit from the utilization of a transgene bioconfinement system. It has been observed that interspecific F_1_*Nicotiana* hybrids (*Nicotiana tabacum* × *Nicotiana glauca*) are sterile and thus proposed that hybrids could be suitable bioconfined hosts for biomanufacturing. We genetically tagged hybrids with green fluorescent protein (GFP), which was used as a visual marker to enable gene flow tracking and quantification for field and greenhouse studies. GFP was used as a useful proxy for pharmaceutical transgenes.

**Results:**

Analysis of DNA content revealed significant genomic downsizing of the hybrid relative to that of *N. tabacum*. Hybrid pollen was capable of germination *in vitro*, albeit with a very low frequency and with significant differences between plants. In two field experiments, one each in Tennessee and Kentucky, we detected outcrossing at only one location (Tennessee) at 1.4%. Additionally, from 50 hybrid plants at each field site, formation of 84 and 16 seed was observed, respectively. Similar conclusions about hybrid fertility were drawn from greenhouse crosses. In terms of above-ground biomass, the hybrid yield was not significantly different than that of *N. tabacum* in the field.

**Conclusion:**

*N. tabacum* × *N. glauca* hybrids show potential to contribute to a bioconfinement- and biomanufacturing host system. Hybrids exhibit extremely low fertility with no difference of green biomass yields relative to *N. tabacum.* In addition, hybrids are morphologically distinguishable from tobacco allowing for identity preservation. This hybrid system for biomanufacturing would optimally be used where *N. glauca* is not present and in physical isolation of *N. tabacum* production to provide total bioconfinement.

## Background

Development of plants as biofactories has progressed since the advent of biotechnology and has rendered the concept of plant molecular farming into existence. Transgene-expression technologies that enable plants to produce large quantities of non-native proteins have useful properties in industrial or pharmaceutical applications, such as production of antibodies, vaccines, and enzymes [1,2]. These technologies have formed the basis of several prospective commercial strategies for biomanufacturing these materials, with advantages of superior economics and ease of scale-up relative to the commonly used microbial and mammalian cell-based fermentation systems [3,4]. Recently the first official clinical-use approval was made for transgenic carrot (*Daucus carota* subsp. *sativus*) cells expressing a human gene for treatment of Gaucher’s disease [5]. This development could be a stepping-stone to using field-grown plants for protein biomanufacturing. Open-field production renders low facilities costs and high scalability, but raises questions of field-based risks. The choice of production platforms (e.g. plant species) is a crucial decision; use of major commodities for plant-made pharmaceuticals (PMP) could warrant extra precaution [6]. Fortunately, there are alternative, non-food species in which the gene-expression technologies are effective. Tobacco (*Nicotiana tabacum*) has been extensively explored as a PMP host [5,7]. However, the possibility of transgene flow to commercial tobacco is a concern. As transgenic varieties of tobacco are not used in any traditional tobacco production, accidental co-mingling of seed or genetic outcrossing, which is low in tobacco (typically less than 5%; [8]), could cause regulatory, legal, or possibly health issues.

There have been several documented examples of unintended gene flow from transgenic plants. In creeping bentgrass (*Agrostis stolonifera* L.), for example, transgene escape to conspecific hosts via pollen and seed can occur over tens of kilometers [9]. ProdiGene, Inc., a former PMP company, was fined by regulatory authorities and was compelled to conduct an expensive clean-up effort for maize PMP volunteers that were detected in a former field site intermingled with soybeans [10]. Such incidents must be avoided if PMPs are to become a commercial reality. There are many possible solutions to the challenge of providing adequate transgene confinement [11]. Harvesting prior to flowering, or manual flower removal might seem obvious and attractive solutions, as this would simultaneously obviate gene flow to other plants via outcrossing and seed formation from the transgenic crop itself. However, the challenge of preventing any flowers from forming in large production acreage necessitates a more reliable system, e.g., bioconfinement.

In this regard, cultivated tobacco and other species in the *Nicotiana* genus provide some potentially useful attributes. Many uncultivated *Nicotiana* species, such as *N. glauca* used in this study, produce biomass yields required for economical leaf-based biomanufacturing. Morphologically, F_1_ hybrids between *N. tabacum* and *N. glauca* are readily distinguishable from cultivated tobacco, have high biomass, and have been reported to be sexually sterile [12-15]. Our own preliminary results from reciprocal crosses between the F_1_ hybrid and its parent species, *N. tabacum* and *N. glauca,* indicated F_1_s were sterile; however when *N. glauca* was the sire, there was low fertility (data not shown). Collectively, these properties lead us to consider interspecific *Nicotiana* hybrids as a potential PMP production platform. PMP constructs could be introduced into the *N. tabacum* and *N. glauca* parents using existing methods. Hybrid seed for field production would then be generated by hybridizing *N. tabacum* with *N. glauca*, an efficient process, as seed yields per plant are very high in these species. Use of these hybrids would provide bioconfinement attributes of (1) production of little or no viable pollen that could transfer transgenes to tobacco production fields, and (2) production of little or no viable seed from the PMP field owing to the lack of viable pollen.

However, the constraints and limitations of F_1_ sterility and production of this system need further research using transgenes to place bioconfinement in the context of biosafety regulations in relevant field settings. A complicating factor is that no regulatory agency has declared specifications for any commercial crop/transgene with regards to field-level gene flow or bioconfinement, although thresholds for presence of transgenic material in conventional food or feed exist. That said, it is safe to assume that bioconfinement should be very high to be effective. Therefore, we set out to assay interspecific hybrid plant pollination of hybrid to tobacco as well as the reciprocal cross. To create a suitable *N. tabacum ♀* × *N. glauca* ♂ hybrid line for these experiments, we transformed both parents with a green fluorescent protein (GFP) marker gene as a proxy for a PMP, providing a convenient way of monitoring transgene flow. DNA content and pollen germination were measured to assess possible differences among multiple hybrid lines and parents. The fertility of the hybrid was characterized by a field gene flow study and by manual crosses in a greenhouse. The productivity of the hybrid, in terms of aboveground green biomass was also determined.

## Methods

### Plants

A summary of plant genotypes used in our studies is listed in Table 1. *N. glauca* was obtained from the US National Plant Germplasm System (NPGS) (plant introduction 307908, accession TW55 from Peru). The following *N. tabacum* lines were obtained from the Kentucky Tobacco Seed Improvement Association, Inc. in Lexington, KY, USA (38°8’N, 84°29’W): *N. tabacum* ‘TN 90’ was from foundation seed lot # 86-02-K-4A. *N. tabacum* ‘MS TN 90’ is a male sterile variety of TN 90 from seed lot # 86-03-KLC-15. *N. tabacum* ‘SN 2108’ is a “dark type” tobacco that is morphologically distinct from the TN 90 cultivar was from seed lot KT D4. Several F_1_ hybrids were used in our studies. The term ‘hybrid GFP’ is used to denote those F_1_s containing a green fluorescent protein (GFP) marker gene. The F_1_ amphihaploid hybrids we produced were the product of unidirectional fertilization of *N. tabacum* with *N. glauca* pollen.

**Table 1:** Plant genotypes used in the studies, including parentage, hybrid and transgene status

**Genotype designation**	**Species or F**_**1 **_**hybrid**	**Transgenic status**	**Marker gene**	**Maternal parent**	**Paternal parent**
TN 90 GFP	*Nicotiana tabacum*	Transgenic	GFP	*Nicotiana tabacum*	*Nicotiana tabacum*
glauca GFP	*Nicotiana glauca*	Transgenic	GFP	*Nicotiana glauca*	*Nicotiana glauca*
hybrid GFP	Hybrid	Transgenic	GFP	TN 90 GFP	Glauca GFP
MS TN 90	*Nicotiana tabacum*	Non-transgenic	N/A	*Nicotiana tabacum*	*Nicotiana tabacum*
SN 2108	*Nicotiana tabacum*	Non-transgenic	N/A	*Nicotiana tabacum*	*Nicotiana tabacum*
HYB BC_1_F_1_	Hybrid	Transgenic	GFP	Hybrid GFP	SN 2108
MS BC_1_F_1_	Hybrid	Transgenic	GFP	MS TN 90	Hybrid GFP
NT-TN 90	*Nicotiana tabacum*	Non-transgenic	N/A	*Nicotiana tabacum*	*Nicotiana tabacum*
NT- glauca	*Nicotiana glauca*	Non-transgenic	N/A	*Nicotiana glauca*	*Nicotiana glauca*
NT- hybrid	Hybrid	Non-transgenic	N/A	*Nicotiana tabacum*	*Nicotiana glauca*

Not all genotypes were used in every experiment.

### Plant transformation

*N. tabacum* ‘TN 90’ and *N. glauca* were transformed with the previously described vector, pBIN mGFP5-ER, which contains the *mGFP5-ER* gene under the control of the constitutive *CaMV 35S* promoter and an *nptII* kanamycin resistance gene [16]. *mGFP5-ER*, a GFP variant, emits green light (λ_max_ = 509 nm) when excited by wavelengths of either ultraviolet (UV) (395 nm) or blue (465 nm) light, which is bright enough to be readily observed under UV excitation and quantifiable with appropriate instrumentation [17,18].

Plants were transformed via *Agrobacterium tumefaciens-*mediated transformation [19]. Leaf explants were prepared by sterilizing young leaves with a mixture of 10% commercial bleach and 0.01% Tween 20, washed with sterile water, then excised into 6 mm^2^ segments. Explants were allowed to soak in a suspension of liquid MS salts containing B_5_ vitamin (DBI medium) and *A. tumefaciens* strain GV3850 harboring the constructs of interest for 30 minutes. Transformed explants were allowed to co-cultivate on solid DBI medium for 48 h prior to transfer to DBI containing Timentin® (400 mg/L) and kanamycin (200 mg/L). Shoots arising from callus were transferred to MS medium containing kanamycin (200 mg/L) for root development [20]. Shoot cultures were grown at 24°C under 16/8 h light/dark periods until rooting occurred. Shoots were then transferred to potting media and acclimated for two weeks. GFP-expressing plants were selected visually with a hand-held longwave UV light (UVP model B-100AP 100 W: 365 nm) as previously described [18]. For further confirmation of the presence of *mGFP5-ER*, genomic DNA was extracted [21] from leaf tissue, and PCR was performed according to Hudson et al. to amplify the full *mGFP5-ER* sequence [22].

T_0_ plants were grown to maturity in 4 L pots in a greenhouse under 16/8 h light/dark periods and corresponding 27°/20°C thermoperiods. Upon flowering, plants were bagged with breathable mesh pollination bags (DelStar Technologies, Inc., Middleton, DE, USA) and manually shaken to promote pollination. T_1_ seeds were collected at maturity and this process was repeated to obtain T_2_ generation seeds. Progeny from selfed T_0_ transgenic events were screened for antibiotic resistance and GFP fluorescence using published methods [23] to confirm transgene integration into hosts.

### GFP *Nicotiana* hybrid production

Our goal was to produce a *Nicotiana* hybrid with sufficient copies of *mGFP5-ER* for tracking purposes, whereby a copy of the *mGFP5-ER* transgene should be present in the genome of each pollen grain. Transformed parent lines were bred to homozygosity for *mGFP5-ER* to the T_2_ generation. Plants were screened for GFP using a handheld UV light to select the brightest GFP-expressing seedlings. GFP expression was then measured by a spectrofluorometer (Fluorolog®-3 HORIBA Jobin Yvon, Edison, NJ, USA) [18,23] and analyzed with software (FluorEssence™ Version 2.5.2.0.HORIBA Jobin Yvon, Edison, NJ, USA) to measure GFP fluorescence. Two strategies were employed to assure homozygosity of each T_2_ line. First, lines were germinated in two flats each and screened with the handheld UV light to determine zygosity of each T_2_ line (using ratios of GFP to non-GFP plants) and inheritance of antibiotic resistance traits among T_2_ lines germination on MS medium containing kanamycin (200 mg/L). Selected T_2_ homozygous lines were designated as “TN 90 GFP” or “glauca GFP” and grown to maturity. These plants were then crossed (TN 90 GFP ♀ × glauca GFP ♂), to produce the sterile hybrid designated as “hybrid GFP” (see Table 1).

#### Estimation of nuclear DNA content

*N. tabacum* has twice as many chromosomes as *N. glauca* (2n = 48 vs 2n = 24), resulting in low likelihood of meiotic chromosome pairing in the F_1_ interspecific hybrid. The absolute DNA content of five hybrid GFP lines and each non-transgenic parental line was estimated by flow cytometry with five replicates each. Plant tissue samples were processed as previously described by Galbraith [24] and analyzed using an Accuri C6 flow cytometer (Accuri Cytometers, Ann Arbor, MI, USA). The known genome size of *Solanum lycopersicum* ‘Roma’, 2C = 1.96 pg [25] was used as an internal standard for estimating DNA content in *N. tabacum* (2C = 9.67), *N. glauca* (2C = 6.91), and the *Nicotiana* hybrid [26].

#### Pollen germination

In order to determine male fertility pollen germination rates of five hybrid GFP lines, wild-type *N. tabacum* (NT-TN 90), and wild-type *N. glauca* (NT-glauca) were compared with five replicates of each. Pollen grains were collected and germinated as previously described [27,28]. Pollen was placed on a microscope slide for observation with an Olympus BX 51 microscope (Olympus Corporation, Shinjuku, Tokyo, Japan) at 100× magnification. A randomly sampled field of view was captured by a digital camera (Olympus Q Color 3) and imaging system. Between 722 and 993 pollen grains were counted for each hybrid line, 1678 grains were counted for non-transgenic (NT)-TN 90 and 1262 grains were counted for NT-glauca. Germination percentage was calculated by dividing the number of germinated pollen grains by the total number of observed pollen grains.

### Field outcrossing

**Figure 1:**
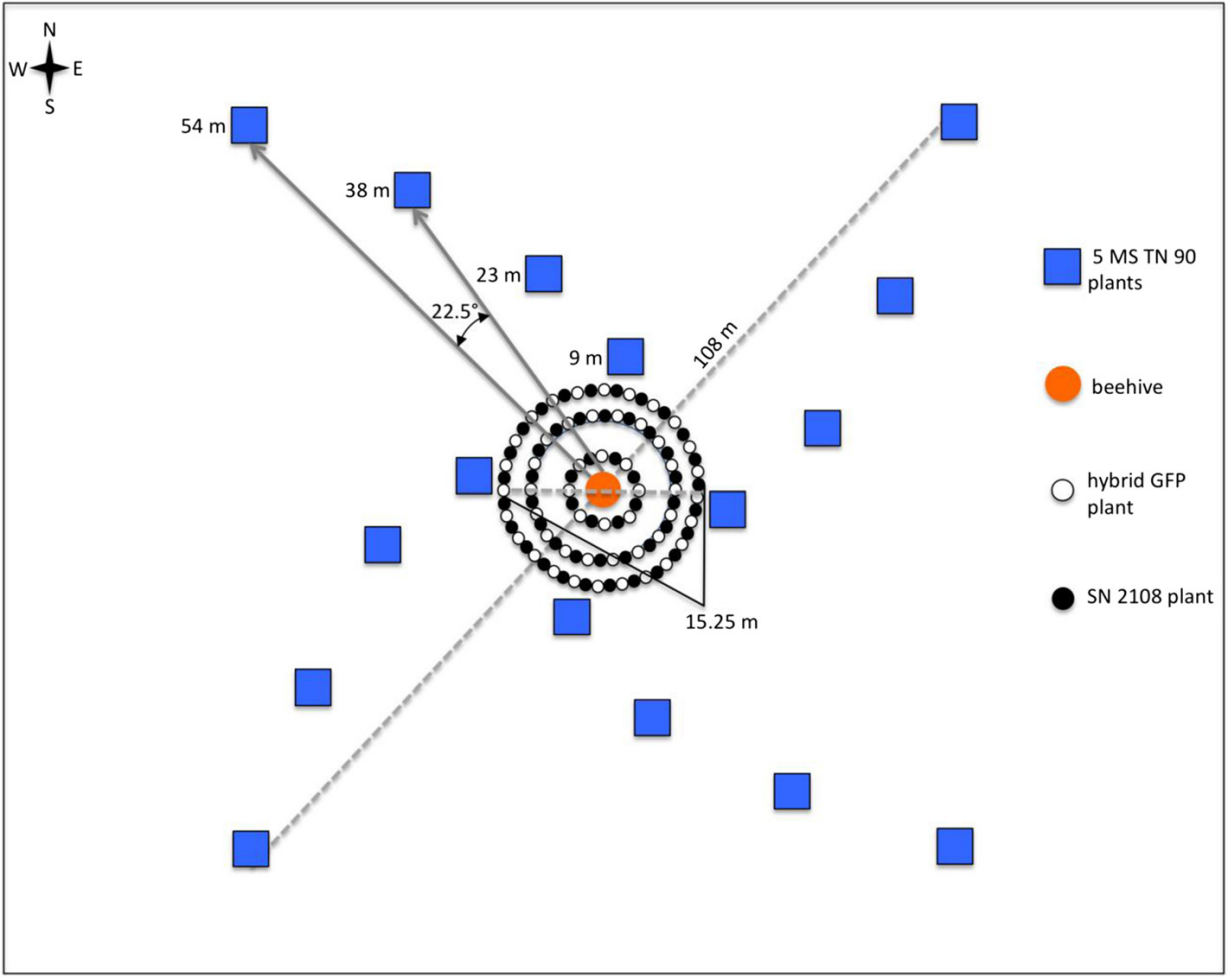
**Design of field gene flow experiment.** A modified Nelder wheel design was used evaluate the gene flow of hybrid GFP plants. Three plant types were used in the experiment: male sterile *N. tabacum* ‘MS TN 90’ were pollen ‘receptor’ plants, hybrid GFP was fluorescently tagged to enable gene flow tracking, and *N. tabacum* type SN 2108 was used as a pollen donor to assure pollen flow was occurring in the field by seed set on MS TN 90 and to test for female sterility of the hybrid GFP plants. A center pollen source patch contained 50 alternating hybrid GFP and fertile SN 2108 plants, spaced approximately 1 m apart. Sixteen 1 m^2^ blocks of male sterile MS TN 90 pollen receptor plants were placed at 9, 23, 38, and 54 m distances from the center and were used to detect pollen via seed formation. Each MS TN 90 plot was 22.5° relative to the adjacent plot as viewed from the center. A honeybee hive was placed at the center of the field site to vector pollen.

Natural outcrossing rates of fluorescently tagged hybrids were estimated in two field experiments conducted at Versailles, Kentucky, USA (38.075784, -84.740575) (KY) and Knoxville, Tennessee, USA (35.891769, -83.959786) (TN). A modified Nelder wheel design (Figure 1) [29] covered approximately 0.931 hectares and contained three plant types. GFP-tagged hybrids and non-transgenic SN 2108 plants were used as pollen donors in the center of the plot, and located along the spokes of the Nelder wheel MS TN 90 plants were used as pollen recipients (Figure 1). The pollen source plot measured approximately 15 m in diameter and contained 3 concentric circles consisting of 50 plants each of alternating hybrid GFP and SN 2108 spaced approximately 1 m apart; a honeybee hive was located at the center of the experiment to ensure pollinator presence. Honeybees were observed on plot flowers. Surrounding this central plot were sixteen blocks of five MS TN 90 plants, each used to detect outcrossing at 9, 23, 38, and 54 m from the center plot in each cardinal direction. Each block of MS TN 90 plants were 22.5° relative to the adjacent block with respect to the center plot in contrast to Nelder’s design where the outer plots are arranged in a linear fashion. This modification was made to take advantage of honeybee flight patterns [30], for flight to and from the center patch. Blocks of MS TN 90 plants and hybrid GFP flowers were monitored throughout the season for formation of pods, which were promptly collected at maturity. SN 2108 plants were used solely as a pollen source and seed set was not of interest because of high self-fertilization rates.

Seed pods were collected at maturity continuously throughout the growing season from both MS TN 90 and hybrid GFP plants and subsequently dried. In Kentucky seed pods were dried after being placed into coin envelopes which were placed into a wooden container with a perforated bottom and shelves. Room temperature air was continuously blown into the bottom of the container until pods were dry. In Tennessee, seeds pods were placed into envelopes and stored in a drying oven at 28°C for 48 hr. After drying, seeds were germinated on filter paper moistened with 0.2% KNO_3_ at alternating 25°C 16 h light: 20°C 8 h dark in accordance with the International Seed Testing Association standards [31]. After three weeks 100 μM GA 4 + 7 was applied to germinate any remaining seed. Seedlings were screened with a handheld UV light to detect GFP-expressing progeny, which were transplanted into potting media and analyzed with a spectrofluorometer as previously described.

#### Pollen tube growth

There could be competition between hybrid GFP and SN 2108 pollen for pollination of MS TN 90 plants in the field. Therefore, pollen tube growth rates were compared. Two plants per line were used, with three replicates per plant measured for 3 d. Pollen was germinated as described above, except 15 μl of BK + medium and pollen was taken every 15 min for 5 h to perform a time-series analysis. Micrographs of pollen tubes were captured and tube lengths were measured against a gridlines on a Hausser Scientific brightline hemacytometer (Horsham, PA, USA). A total of 8750 pollen grains were observed from SN 2108 plants and 7,524 grains were observed from hybrid GFP.

### Manual crosses

Manual crosses were conducted in a greenhouse in Lexington, KY, and were performed to mirror the possible crosses expected in the field experiment. To determine the outcrossing capability of the hybrid, hybrid GFP plants were crossed with MS TN 90. To evaluate the seed setting capacity of the hybrid, SN 2108, the pollen source plant type used in the field experiment were crossed to hybrid GFP plants. Hybrid GFP plants were also intercrossed to determine any transgene event reproduction variability. In addition, both fertile plants, SN 2108 and MS TN 90, were crossed as controls. Pollen-recipient flowers were emasculated prior to crossing. A total of 96 crosses were performed for (hybrid GFP ♀ × SN 2108 ♂) crosses and 95 crosses were performed for (MS TN 90 ♀ × hybrid GFP ♂). Ten crosses were performed between a pair of MS TN 90 ♀ × SN 2108 ♂ plants and also for the pair of crossed hybrid GFP plants. Seeds derived from crosses containing GFP-tagged plants were germinated, transplanted to potting mix, and analyzed with a spectrofluorometer as previously described.

### Aboveground biomass

#### Field study

Vegetative biomass of NT-TN 90 and NT-hybrid plants were measured. The study employed a complete randomized block design with three replications. Seeds were germinated in float trays and transplanted at a density of approximately 12,000 plants/hectare. Plots consisted of four 6 m rows containing 80 plants. Drip irrigation was employed for supplemental watering. Harvesting occurred during the budding stage by trimming rows to 4.6 m for standardization and measuring all green biomass above the 30 cm mark above the soil line. Subsequent harvests occurred every 28 to 35 days for a total of three harvests and the study was repeated in two consecutive years.

#### Greenhouse study

To determine the productivity of hybrid GFP relative to the parent lines NT-hybrids, five replicate plants from hybrid GFP, glauca GFP*,* TN 90 GFP, and NT-hybrid lines were grown. A completely randomized design was used to determine fresh biomass productivity. For each replicate, approximately 10 seedlings were germinated in a 4 L pot in a greenhouse, culled down to one plant 2 weeks post-germination and transplanted to a 12 L pot 2 months post-germination. Plants were grown under a 16/8 h light/dark periods and corresponding 27°/20°C thermoperiods and spaced at 1 m centers. Plant productivity was analyzed by measuring fresh weight harvested at the budding stage 30 cm above the soil line. Plants were allowed to re-grow to the budding stage for two measurements.

### Statistical analysis

All analysis of variance (ANOVA), regression, contingency table analyses, and chi-squared tests were performed using SAS (Version 9.3 SAS Institute Inc, Cary, NC, USA) with a significance level of p < 0.05. Proc mixed was used for all ANOVA calculations. The least significant difference was used for mean separations if ANOVA results were found to be statistically significant. Log and rank transformations and were used when data did not meet the assumptions of a normal distribution by the Shapiro-Wilk test [32] or equal variance by the Levene test [33].

## Results

### Transformation and characterization of parental lines and hybrids

Multiple transgenic events from *N. tabacum* ‘TN 90’ and *N. glauca* were generated. T_1_ plants that highly expressed GFP and were selected and self-pollinated. Homozygosity of T_2_ lines was confirmed by progeny analysis by antibiotic screening on media and GFP expression. The homozygous T_2_*N. tabacum* and *N. glauca* lines were crossed to produce hybrid GFP lines, where GFP was visible in the stems and leaves of the plants.

**Figure 2:**
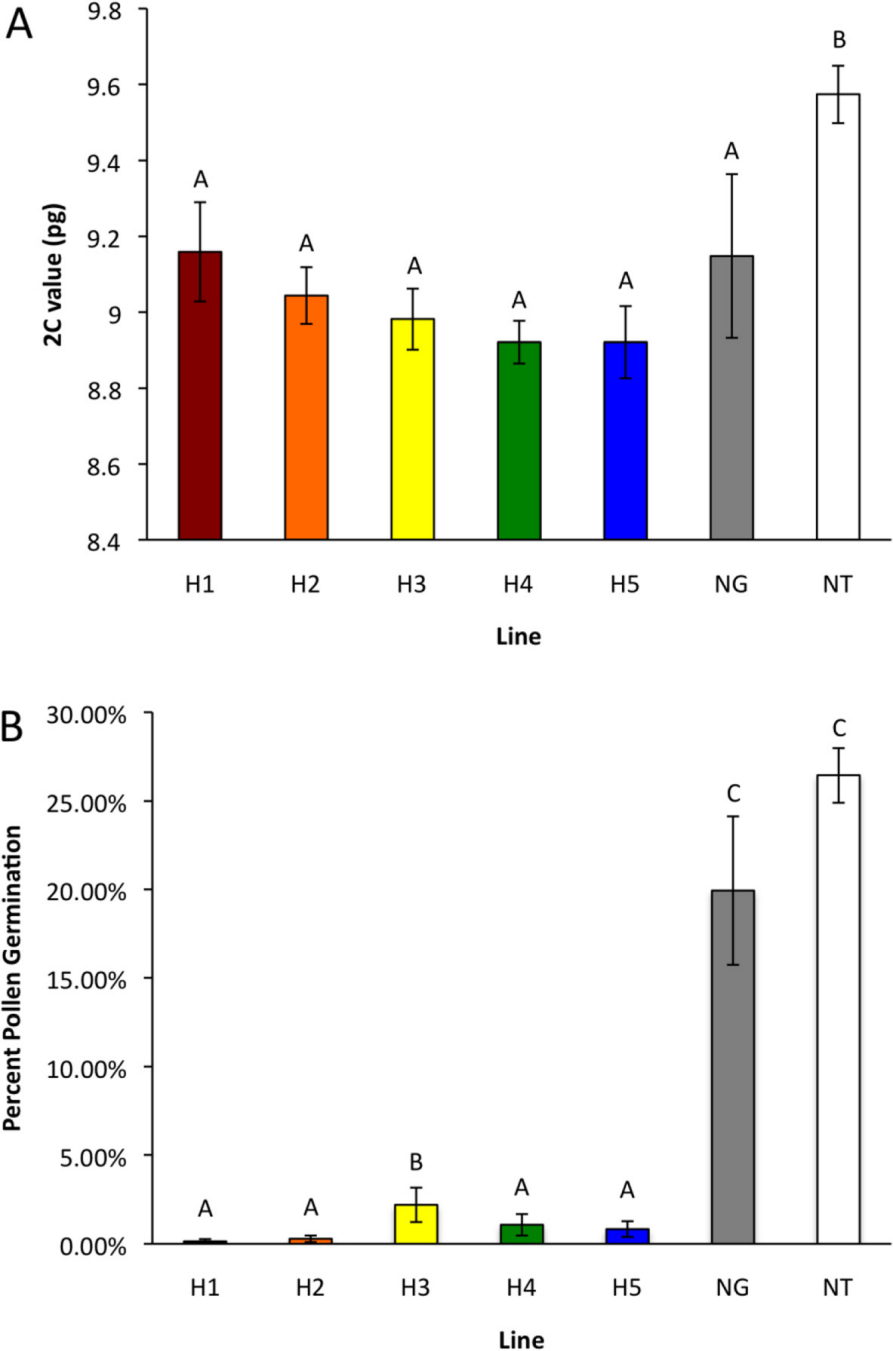
**DNA content and pollen germination analysis. (A)** DNA content was estimated by flow cytometry and **(B)** pollen viability was estimated by pollen germination. Hybrid GFP-plant lines: H1. H2, H3, H4, and H5 and non-transgenic parent lines NT-glauca (NG) and NT-TN 90 (NT) n = 5. Mean separation is by Fishers LSD and bars marked by the same letter are not significantly different (p < 0.05). Error bars are the standard error of the means.

To assess the effectiveness of the sterile hybrid system as a bioconfinement platform, it was important to characterize hybrid-line variation in DNA content and male fertility. The absolute DNA content of the five hybrid GFP lines did not differ significantly from each other or the paternal line *N. glauca*, but differed from the maternal line *N. tabacum* (p = 0.010) (Figure 2A). Although pollen germination differed across hybrid and parent lines (p < 0.001), only one hybrid line differed from the other four with a higher percentage of germination (Figure 2B). Regression analysis revealed no association between pollen germination and DNA content at p = 0.05.

### Field outcrossing experiments

Preliminary hand-crosses resulted in extremely low fertility of the interspecific hybrid plants, which prompted us to assay natural outcrossing in the field. We observed no aberrations of pod formation and seed-set on the MS TN 90 plants in each field experiment. No GFP-positive seedlings were found after germination of 7,340 MS TN 90 seeds from the KY field site, however, one single GFP positive seedling (collected from a distance of 9 m from the field center) was found from 74 germinated seeds from the TN field site (a 1.4% outcrossing rate) (Table 2). The sole survivor died several weeks after germination. In the testing of our initial hypothesis that there would be no hybrid GFP outcrossing, a Pearson’s chi squared test indicated a nonsignificant deviation from the null hypothesis (p < 0.05) with p = 1.0 and p = 0.9 for KY and TN field sites, respectively. Whereas pollen movement occurred in these fields there was nearly no transgene ‘outflow’ from the hybrids.

**Table 2:** Summary of seed collection, germination, and analysis from hybrid GFP and MS TN 90 plant at the Kentucky and Tennessee field sites

**Site**	**Kentucky**		**Tennessee**	
**Plant**	**Hybrid GFP**	**MS TN 90**	**Hybrid GFP**	**MS TN 90**
Total pods collected	73	155	263	118
Blocks setting seed^a^	N/A	15	N/A	9
Plants setting seed	5	N/A	11	N/A
Seeds collected	16	11170	84	5968
Seeds germinated	9	7340	38	74
Total surviving seedlings	5	7340	32	73
GFP positive seedlings^b^	4	0	31	1
GFP negative seedlings^b^	1	7340	1	74
Surviving GFP positive seedlings confirmed with spectrofluorometer	4	0	31	0

^**a**^*N. tabacum* ‘MS TN 90’ plants were planted and harvested in blocks of five plants.

^b^Presence of green fluorescent protein was confirmed visually with a handheld UV light.

All pods formed on the hybrid GFP plants were examined; only 16 seeds were derived from 5 plants at the KY site and 84 seeds formed from 11 plants at the TN field site. A total of 47 of these seeds from both sites germinated, yielding 37 seedlings and hence mature plants. Thirty-five seedlings were GFP-positive, both by observation under UV light and by spectrofluorometric analysis (Table 3). These observations suggest that while seeds containing the transgene can be formed on the hybrids in an open-pollination environment, the total seed-set on these plants is very small in comparison with that of conventional *N. tabacum*. Moreover, none of the progeny grown from the very limited hybrid GFP seed production in the field experiment produced any seed when self-pollination was attempted.

**Table 3:** Results of crosses made in the greenhouse to examine the sexual compatibility of hybrid GFP plants and the fertility of their progeny

**Genotypes**^**a**^	**Plants crossed**^**b**^	**Total crosses**^**c**^	**Crosses resulting in seed production**	**Total seed count**	**Seeds that germinated**	**Surviving plants**
(A) F1 crosses						
(hybrid GFP × SN 2108)^d^	12:12	95	7	12	5	3
(MS TN 90 × hybrid GFP)^e^	12:12	96	9	445	2	2
(hybrid GFP × hybrid GFP)	1:1	10	0	0	0	0
(B) Backcrosses						
(MS TN 90 × HYB BC_1_F_1_)	2:1	60	0	0	0	0
(MS BC_1_F_1_ × NT-glauca)	1:1	20	2	128	74	74
(MS BC_1_F_1_ × NT-TN 90)	1:1	20	0	0	0	0
(MS BC_1_F_1_ × hybrid GFP)	1:1	20	0	0	0	0

^a^Crosses listed by (female ♀ × male ♂).

^b^Refers to numbers of each plant used in crosses with respect to the plant order in the cross column.

^c^A ‘cross’ is constituted by a pollen transfer from one plant to the flower of another plant.

^d^Progeny of this cross formed the individuals named HYB BC_1_F_1._

^e^Progeny of this cross formed the individuals named MS BC_1_F_1_.

Progeny generated from the initial hybrid GFP crosses were used in a subsequent set of crosses to test sexual compatibility of potential volunteers with other *Nicotiana*s. The crosses shown in the top-most rows each for (A) F_1_ crosses and (B) Backcrosses, produced progeny that were subsequently used for additional crosses. See Table 1 for plant nomenclature.

### Pollen tube growth: pollen competition experiment

To estimate the degree of competition from hybrid GFP and SN 2108 to pollinate MS TN 90 plants in the field experiments, pollen tube growth was measured. Simple linear regression of the SN 2108 pollen samples that germinated revealed that pollen tube length and time was positively correlated (R^2^ = 0.203) with an average growth rate of 0.043 ± 0.005 mm per 15 minutes. For hybrid GFP plants, linear- or polynomial regression models did not reveal any association of pollen tube length and time (p = 0.206), thus pollen competition might have occurred between plant types.

### Manual crosses

Crosses were performed under greenhouse conditions among the transgenic hybrid GFP lines, MS TN 90, and SN 2108 (Table 3). We observed robust seed production from the MS TN 90 by SN 2108 cross (data not shown). However, the pollination of hybrid GFP plants by SN2108 resulted in few seeds from 7 of 96 crosses (Table 3). Five of these seeds germinated (71% viability) but only 3 plants survived to maturity. Testing the outcrossing potential of the hybrid GFP to MS TN 90 resulted in seed from 9 out of 96 crosses. Viability of this seed was extremely low (< 0.7%); just 3 out of 445 seeds germinated (Table 3), and one plant died shortly after germination. Attempted crosses between two hybrid GFP plants resulted in no seed.

Subsequently, crosses were performed to determine fertility of the progeny derived from the hybrid GFP ♀ × SN 2108 ♂ cross, designated as HYB BC_1_F_1_ and the MS TN 90 ♀ × hybrid GFP ♂ cross, designated as MS BC_1_F_1_ (Table 3). This germplasm, in effect, simulates the possible fates of volunteer plant populations as a result of comingling of genetic material from a hybrid GFP field and nearby *Nicotiana*s. HYB BC_1_F_1_ pollen was crossed to MS TN 90 plants to test if a volunteer produced from seed set on hybrid F_1_ plants could pollinate a neighboring *N. tabacum* field; none of the 60 crosses set any seed. MS BC_1_F_1_ plants, the result of the few successful hybrid GFP outcrossing to MS TN 90, were pollinated with non-transgenic *N. glauca* and TN 90 pollen, and hybrid GFP pollen. Only the MS BC_1_F_1_ ♀ × NT-glauca crosses produced any seed, confirming earlier findings that *N. glauca* can successfully pollinate hybrids*.* No other crosses produced seed.

These results suggest that the small amount of viable seed formation that could occur in field production of the hybrids via pollen introduced from within (hybrid plants) or outside that field (conventional tobacco) would not result in the persistence of transgenic plants in that field beyond one generation. The only exception to this limited persistence would be if *N. glauca* plants grew inside or adjacent to a hybrid field site.

### Above-ground biomass

**Figure 3:**
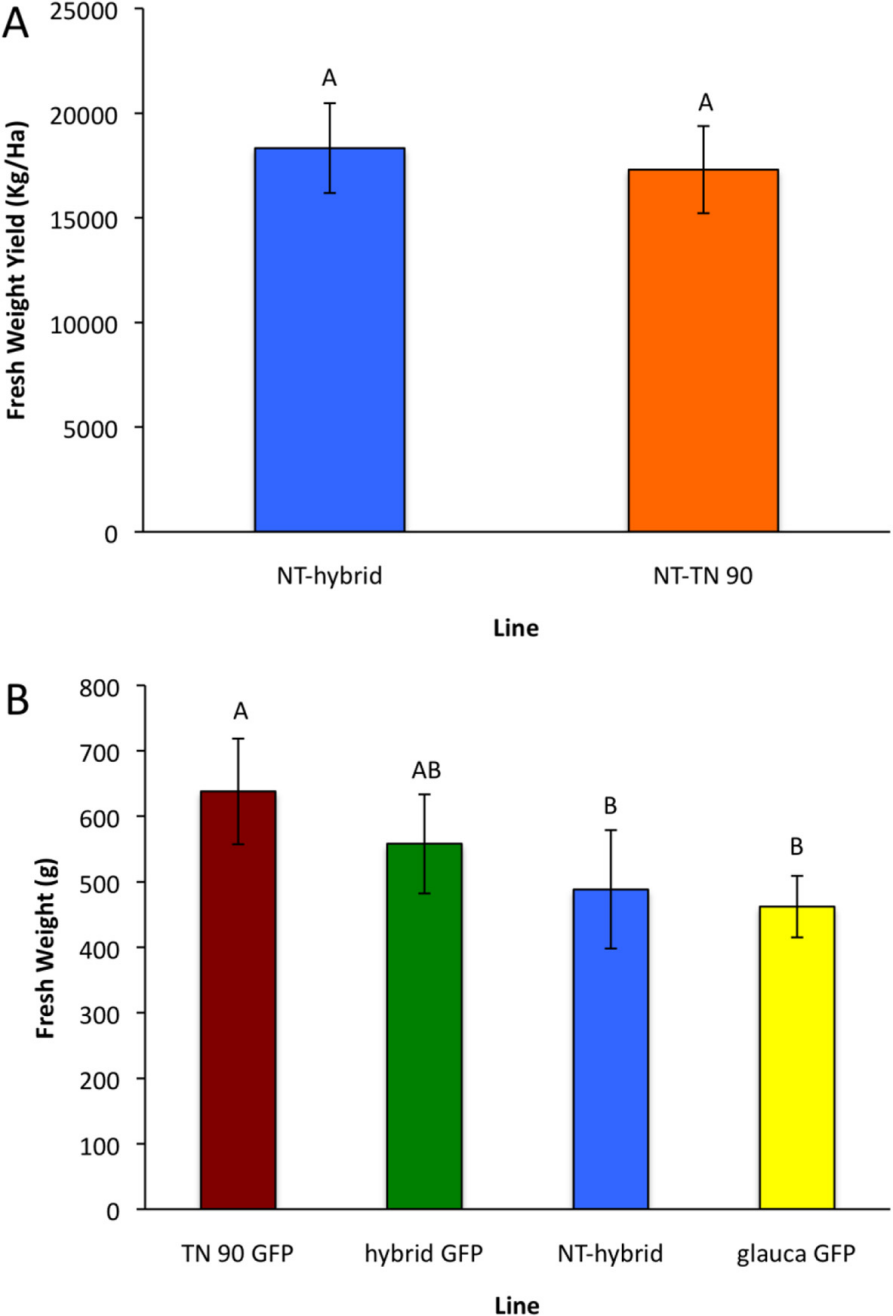
**Fresh aboveground biomass productivity. (A)** Productivity was measured for nontransgenic hybrid plants and nontransgenic TN 90 plants in a field experiment over two years with three measurements per year. **(B)** A greenhouse study was conducted during one year with two measurements of the hybrid GFP line, the transgenic parental lines of the aforementioned hybrid, and a nontransgenic hybrid line*.* Mean separation is by Fishers LSD and bars marked by the same letter are not significantly different (p < 0.05). Error bars are the standard error of the means.

For interspecific hybrid *Nicotiana*s to have potential as production-host plants in biomanufacturing they should produce high biomass; e.g., comparable to commercial tobacco. There were not significant differences of biomass between hybrids and TN 90 in the field experiment (p = 0.738; Figure 3A). There were biomass differences among plant types in the greenhouse study (Figure 3B). We conclude that interspecific hybrid tobacco performed comparably to *N. tabacum* in biomass.

## Discussion

Tobacco has several qualities that make it suitable for use as a production platform for biomanufacturing. It is easily transformable to achieve a high content of soluble heterologous protein, and accommodates a range of alternative gene-expression systems including viral transfection, transient expression via *Agrobacterium* vectors, and stable nuclear and chloroplast transformation methods [34]. The tobacco system is very productive; the above-ground portion of the plants can be harvested several times per growing season, producing up to 25 tonnes ha^-1^ of biomass [35]. In addition, tobacco has a track record of experimental use as a bioreactor for producing vaccines, antibodies, and cytokines [36-38], and its high biomass yields and prolific production of seed suggest efficiency and flexibility of scale-up. Tobacco is being used in biomanufacturing in the private sector currently. Planet Biotechnology has a dental caries product in Phase II clinical trials and Medicago, Inc. has an influenza vaccine in Phase I clinical trials. The interspecific hybrid (*N. tabacum ♀* × *N. glauca ♂*) has been described as an effective production platform for an animal vaccine [36]. The ‘sterility’ of this hybrid has been cited as a distinct benefit for production in the environment over tobacco [36] contributing to the desired goal of complete bioconfinement of heterologous pharmaceutical genes and proteins in biomanufacturing applications, but until now this attribute has not been evaluated in detail.

Previous studies on the fertility of the *Nicotiana* interspecific F_1_ hybrids concluded that they were infertile [36]; Trojak-Goluch and Berbec described the F_1_ amphihaploids resulting from pollinating *N. tabacum* with *N. glauca* as “completely self- and cross-sterile” [12,14,36]. Al-Ahmad et al. observed their *N. sylvestris* × *N. tabacum* hybrids were incapable of self-pollination or of successfully generating any progeny when backcrossed to the female parent *N. sylvestris*[15]. The principal difference between our findings and those earlier studies is our observation of occasional, albeit very minimal, fertility of *N. tabacum ♀* × *N. glauca ♂* hybrid plants, both in the greenhouse and in the field. Similarly the germination rate of pollen formed on different (transgenic) hybrid individuals was very low but measurable and variable (Figure 2B), contrasting with the observation from Ling et. al. that the pollen from transgenic *N. tabacum ♀* × *N. glauca* ♂ hybrids was non-viable [36]. This disparity is important in relation to the goal of obtaining optimum bioconfinement, as it cautions that the ‘sterility’ trait may not be sufficiently predictable for this hybrid host to be the sole strategy employed.

Differences in residual fertility among hybrid plants may have resulted from variation among transgenic events following fusion of the gametes owing to amphiploidy effects. Trojak-Goluch and Berbec concluded that meiosis in pollen mother cells was mostly asynaptic, based on the number of univalent chromosomes, which varied from cell to cell during metaphase I; they also noted that chromatid bridges, lagging chromosomes and a lack of one of the meiotic divisions were common observations during anaphase and telophase I [12]. Hence we were interested in variation of total DNA content among types, especially among hybrids. The absolute DNA content of NT-TN 90 was similar to previously reported values for *N. tabacum*[26,39] (Figure 2A). Also, our estimations for *N. glauca* reflected a wide range of previously reported 2C values [26,40]; Figure 2A). The additive 2C value of the parental lines of the hybrid was 18.72 pg, but the average 2C value of hybrid GFP ranged from 8.92 pg to 9.16 pg among five lines, indicating a substantial genome downsizing of 50% (Figure 2A). The DNA content of these hybrid GFP plants, which are amphiploid/tetraploid (3n = 2× = 36) [14], were not significantly different from that of NT-glauca (2n = 2× = 24). Polyploids within the genus *Nicotiana* have been reported to exhibit increased DNA content or to show additivity in DNA content relative to the sum of the diploid parents’ values, and genome downsizing is a common occurrence in many plant species [41]. One might be concerned that downsizing during the hybridization process could compromise subsequent transgene expression levels. However, we consistently observed that F_1_ hybrid GFP, which was produced from parent lines homozygous for *mGFP5-ER*, produced GFP-expressing progeny in subsequent crosses, and therefore apparently retained multiple copies of the *mGFP5-ER* transgene.

Even with the extremely limited fertility that we observed, the interspecific hybrid strategy does represent a bioconfinement improvement over expressing genes in *N. tabacum* in the context of bioconfinement. The manual plant crosses showed low production of viable seeds: from *N. tabacum* type SN 2108 to the hybrid, and from hybrid to *N. tabacum* MS TN 90 (Table 3). This finding was confirmed in the field setting, as evidence of GFP presence in the *N. tabacum* MS TN 90 progeny was found among thousands of seeds (Table 2). In contrast, a very small number of viable seeds were set on the hybrids in the field (Table 2). However, we note that the Tennessee field study data are very limited owing to very low seed germination; we cannot explain this finding.

The use of the interspecific hybrid host for biomanufacturing should not be considered to be a failsafe bioconfinement solution. A male-sterility trait could be employed in *N. tabacum* to improve bioconfinement. Although, cytoplasmic male sterility (CMS) sometimes suffers from reversion of the phenotype [42,43], CMS might also be useful for bioconfinement. However, if the hybrid is used in isolation from tobacco production fields (say, 10 km), AOSCA seed production regulations state only 0.40 km of isolation of different fertile tobacco cultivars the combination of partial bioconfinement in the hybrid system and physical isolation could be sufficient to mitigate risks.

Of the crosses that were attempted, the most productive was the pollination of the hybrid by *N. glauca* (data not shown). *N. glauca* is currently present in twelve U.S. states [44], including regions in which tobacco agriculture occurs (e.g. Ohio). A regulatory process examining the hybrid for use in outdoor production of recombinant proteins would need to evaluate wild *N. glauca* distribution and proximity to PMP-production fields. However, the pollination of *N. glauca* by the hybrid was consistently unsuccessful in our hands (data not shown), which is encouraging in relation to concerns about transgene transfer to the wild species in the environment.

## Conclusions

The extremely low fertility of interspecific *Nicotiana* hybrid plants could contribute to an effectively bioconfined biomanufacturing platform, which would also likely require physical isolation from commercial tobacco production as well as from wild *N. glauca*. The hybrid progeny obtained from pollinating *N. tabacum* using the uncultivated species *N. glauca* represent a good candidate for a bioproduction host since it has extremely low fertility and sufficient above-ground biomass in the field.

## Competing interests

The authors declare that they have no competing interests.

## Authors’ contributions

JHR: Performed all plant transformation experiments, carried out the field experiment in Tennessee, performed pollen germination experiments, DNA content analysis, greenhouse biomass studies, and drafted the document: REM: Conceived portions of the study, assisted in the field design, bred the original interspecific hybrid, produced the experimental hybrids, performed manual greenhouse crosses, and carried out biomass and gene-flow field experiments in Kentucky. RJM: Coordinated the study and assisted with analysis. ODC: Conceived portions of the study and assisted with coordination and execution of the study. CNS: Conceived portions of the study, coordinated the study and assisted with revisions HMD: Conceived portions of the study, coordinated the study and provided critical review. All authors contributed to and approve the final text.
